# Isolation and evaluation of the efficacy of bacteriophages against multidrug-resistant (MDR), methicillin-resistant (MRSA) and biofilm-producing strains of *Staphylococcus aureus* recovered from bovine mastitis

**DOI:** 10.1186/s12917-022-03501-3

**Published:** 2022-11-17

**Authors:** Fatemeh Mohammadian, Hamideh Kalateh Rahmani, Behnam Bidarian, Babak Khoramian

**Affiliations:** 1 Department of Clinical Sciences, Faculty of Veterinary Medicine, Ferdowsi University of Mashhad, P.O. Box: 9177948974, Mashhad, Khorasan Razavi Province Iran; 2 Department of Pathobiology, Faculty of Veterinary Medicine, Ferdowsi University of Mashhad, Mashhad, Iran

**Keywords:** Bacteriophage, *Staphylococcus aureus*, Bovine Mastitis, Alternative treatment, *Podoviridae*, Hard-to-treat

## Abstract

**Background:**

*Staphylococcus aureus* (*S. aureus*) is one of the major causes of bovine mastitis with significant economic losses around the worldwide. The emergence of multidrug-resistant (MDR), methicillin-resistant (MRSA) and biofilm-producing strains of *S. aureus* challenges the treatment strategies based on the antibiotic application. Today, alternative or combinational treatment options such as bacteriophage application has received much attention. The goal of the present study was to focus on isolation and evaluation of the efficacy of bacteriophages with specific lytic activity against *S. aureus* strains with low cure rates (MDR, MRSA and biofilm-producing strains).

**Results:**

In the present study, two phages belonging to the *Podoviridae* family with specific lytic activity against *S. aureus* were isolated from the sewage of dairy farms and designated as *Staphylococcus* phage M8 and *Staphylococcus* phage B4. Latent period and burst size for *Staphylococcus* phage M8 (70 min, 72 PFU/cell) and *Staphylococcus* phage B4 (30 min, 447 PFU/cell) were also defined. Our results revealed the susceptibility of MDR (4/20; 20%), MRSA (4/13; 30.8%) and biofilm-producing (1/10; 10%) strains to *Staphylococcus* phage M8. Moreover, one biofilm-producing strain (1/10; 10%) was susceptible to *Staphylococcus* phage B4. Furthermore, both phages kept their lytic activity in milk. They reduced the *S. aureus* population by about 3 logs in cultured milk after 8 h of incubation.

**Conclusion:**

In conclusion, it seems that both phages had the potential to serve as biological control agents alone or in combination with other agents such as antibiotics against infections induced by *S. aureus*. However, further studies are needed to investigate the efficacy of these phages in vivo.

## Background

Mastitis is a mammary gland inflammation caused by a variety of infectious and non-infectious agents that affects a large percentage of dairy cows worldwide and estimated to be the most expensive disease affecting dairy cows. *Staphylococcus aureus* (*S. aureus*) is one of the major causes of bovine mastitis with significant economic losses [1]. Antibiotic therapies are frequently ineffective in treating *S. aureus* infections, due to some of the unique features of the pathogen such as: the ability of the organism to colonize and produce micro-abscesses in the mammary gland which leads to be protected from normal defense mechanisms, potential of invading bovine mammary epithelial cells, switching to the small-colony variant (SCV) phenotype and biofilm formation which are relevant to chronic and recurrent infections, the capability of persistent within phagosomes, converting to L-form when exposed to antibiotics, and the ability to produce toxins [2–4].

Because of low clearance rate in *S. aureus* mastitis, procedures have been developed to continue treatment for 6 to 8 days to maintain therapeutic levels of antibiotics. Therefore, the overuse of antibiotics has resulted in the rise of multidrug-resistant *S. aureus* (MDR) and methicillin-resistant *S. aureus* (MRSA) [5]. It is well established that antimicrobial use is the main driver of antibiotic resistance. As an example, the overuse of antibiotics and antibiotic prescribing inconsistent with antibiograms gives the opportunity to multiply resistant bacteria such as MRSA by destroying the dominant and sensitive clones of bacteria [6]. In addition to the emergence of MDR and MRSA strains of *S. aureus*, treatment of biofilm-producing strains is indeed a challenge. Biofilm structure protects bacterial pathogens against harsh conditions such as effective levels of antibiotics and host immune defense mechanisms. Today, it is believed that the ability of biofilm-formation by a pathogen is connected to recurrent and chronic mastitis [7, 8]. Consequently, searching to find an alternative or combinational treatment options has been developed. One such alternative is the bacteriophage (phage) application that has received much attention during the last decade [9–12].

Bacteriophages are bacterial viruses that attach to and kill their hosts by internal propagation and consequent bacterial lysis. Phages act specifically and cells except their bacterial host are safe from attack. Unlike antibiotics, phages selectively kill target bacteria while leaving the hosts normal microflora intact, preventing bacterial dysbiosis and subsequent infections. Thus, the application of phages to inhibit bacterial growth could be a natural, harmless, and effective alternative to antibiotics [13]. Bacteriophages can be categorized into four types according to their infection strategies: a) lytic and non-temperate, b) chronic and non-temperate, c) lytic and temperate, and d) chronic and temperate. For therapeutic purposes, “professionally lytic” phages (category a) which are obligately lytic, do not belong to the temperate phages and are not recent lytic mutants of the temperate phages, should be used [14].

Phages can be characterized by their physiological traits. Among these characteristics, latent period (the time taken by phage to reproduce inside an infected host cell) and burst size (the number of newly synthesized phage particles from an infected cell) are important infection parameters of lytic phages that can be determined by analysis of the one-step growth curve [15]. Moreover, phages are classified based on their morphological and genomic aspects [16].There are several phages families; however, most phages belong to one of the three following families: *Myoviridae*, *Siphoviridae* and *Podoviridae* [17].

There is limited information about lytic activities of phages, especially against MDR, MRSA and biofilm-forming strains of *S. aureus*, in veterinary medicine compared with medicine. The majority of studies on lytic phages of *S. aureus* in veterinary medicine (especially in mastitis) have thus far focused on phage isolation in general, and hard-to-treat phenotypes of the bacteria such as MDR, MRSA and biofilm-producing strains are neglected. For example, Synnott et al., (2009) isolated two anti-staphylococcal phages include: SA039 and SA012 from sewage influent, and examined their activity against 15 *S. aureus* strains isolated from bovine mastitis milk. Ultimately, consequences showed that 13 strains were susceptible to phage SA039 and eight strains were susceptible to phage SA012 [18]. Moreover, in 2013, 10 newly isolated phages (Ufv-aur2 to Ufv-aur11) were introduced by Dias et al. It was revealed that phages 2, 5, 6 and 11 had strong lytic activities against mastitis-causing *S. aureus* strains which were mainly resistant to penicillin and ampicillin [19]. In both mentioned studies, there was no focus on treating hard-to-treat phenotypes of *S. aureus* by phages. Therefore, in the present study we placed a great emphasis on isolation and evaluation of the efficacy of bacteriophages with specific lytic activity against *S. aureus* strains with low cure rates (MDR, MRSA and biofilm-producing strains).

## Results

### Bacteriophage isolation and titration

**Fig. 1:**
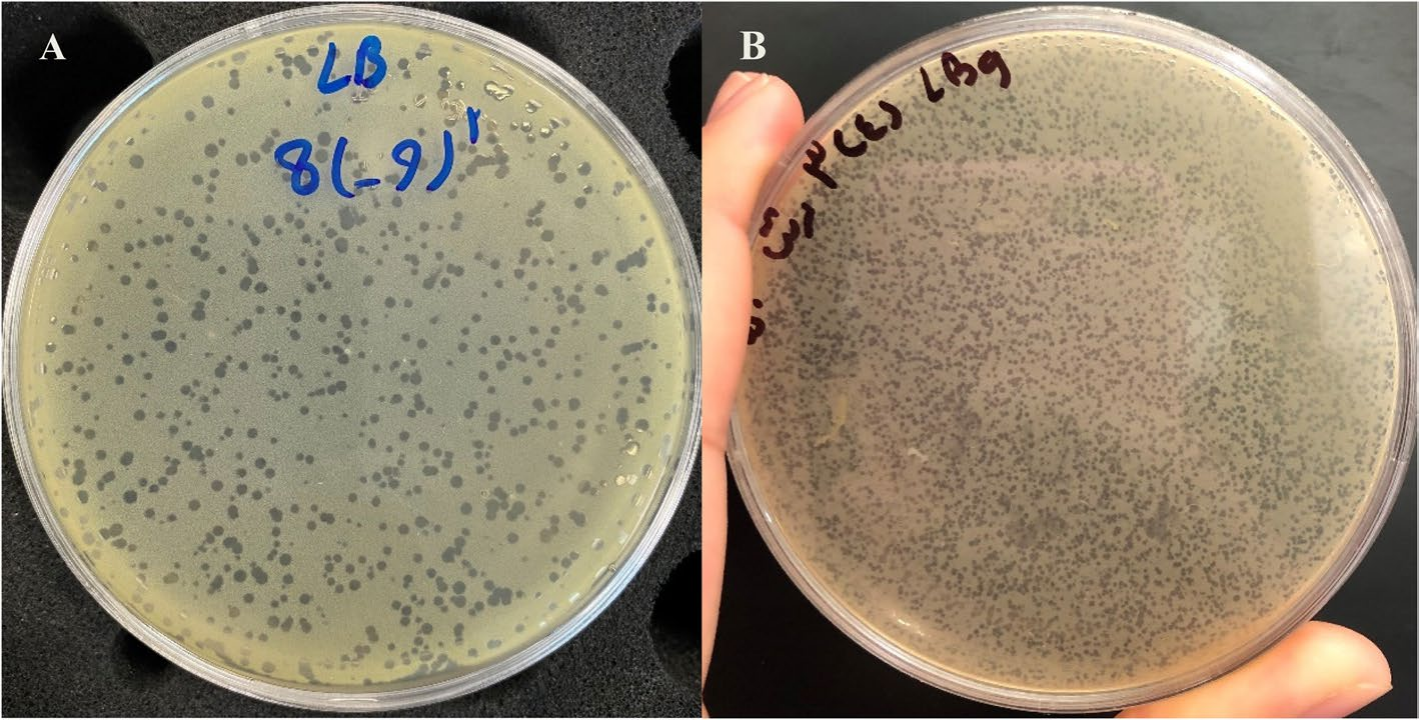
DLA plates of *staphylococcus* phage M8 (**A**) and *staphylococcus* phage B4 (**B**)

**Fig. 2:**
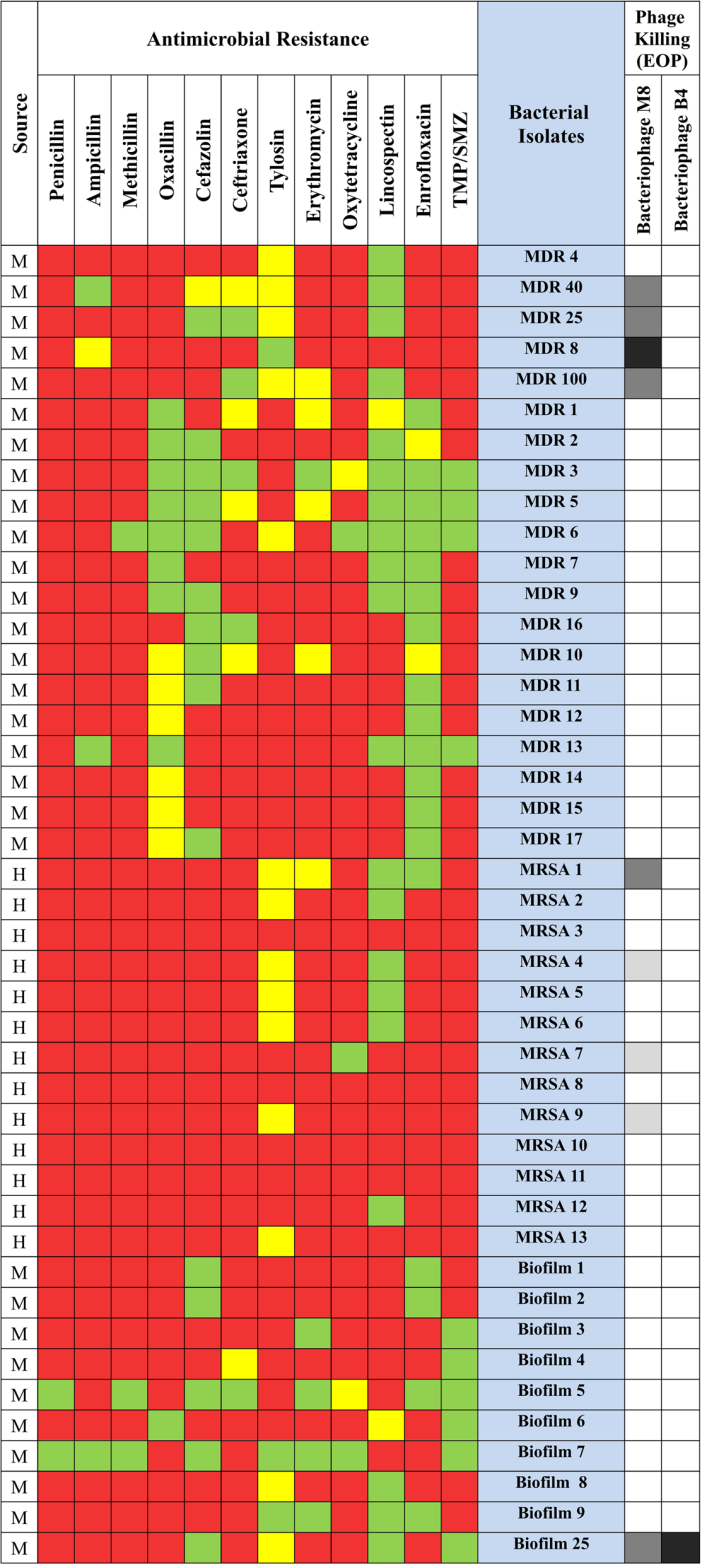
Antibiotic susceptibility and Phage Killing of *S. aureus* isolates. Source: M = Mastitis, H = Human; Antimicrobial Resistance: Green = Susceptible, Yellow = Intermediate, Red = Resistant; Phage Killing (EOP): Black = EOP equal to 1, Dark Gray = 0.001 ≤ EOP ≤ 0.999, Light Gray = EOP < 0.001, White = No Growth

In the current study, two phages with specific lytic activity against *S. aureus* were isolated and purified from six sewage samples of dairy farms and designated as *Staphylococcus* phage M8 and *Staphylococcus* phage B4 (Fig. 1). The host strain for *Staphylococcus* phage M8 was an MDR strain with resistant pattern to enrofloxacin, tetracycline, oxacillin, lincomycin, erythromycin, cefazolin and ceftriaxone. A biofilm-forming *S. aureus* isolate was a host strain for *Staphylococcus* phage B4 as well (Fig. 2). The titers for *Staphylococcus* phage M8 and *Staphylococcus* phage B4 were determined 1 × 10^9^ and 1 × 10^8^ PFU/mL, respectively.

### Host range determination

**Fig. 3:**
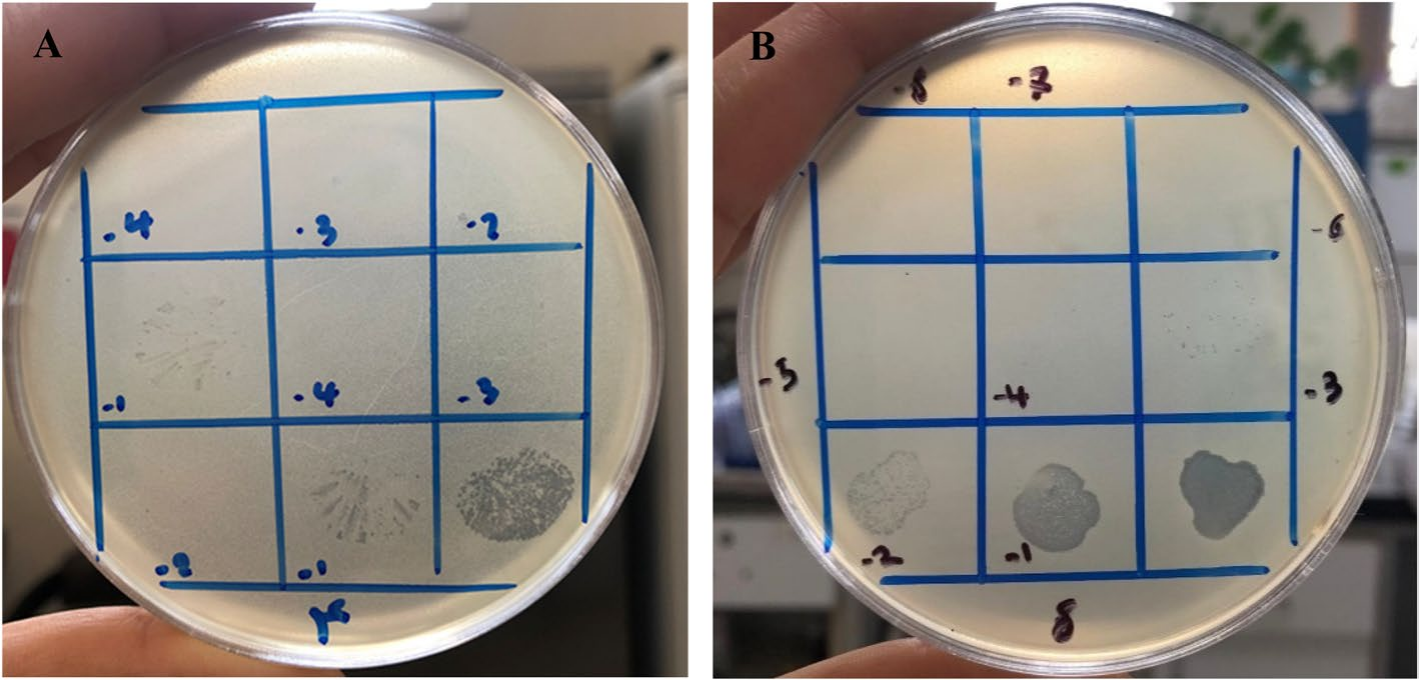
EOP bacterium strain number 4 susceptible to phage B4 (**A**), and bacterium strain number 8 susceptible to phage M8 (**B**)

Using the EOP approach, the effects of isolated bacteriophages on various bacterial strains were examined (Fig. 3). In general, among 43 *S. aureus* strains (13 MRSA, 20 MDR and 10 biofilm-producing strains) 9 isolates (20.9%) were sensitive to *Staphylococcus* phage M8 and one isolate (2.3%) was sensitive to phage *Staphylococcus* phage B4 (Fig. 2). Regarding details, our results revealed the susceptibility of MDR (4/20; 20%), MRSA (4/13; 30.8%) and biofilm-producing (1/10; 10%) strains to *Staphylococcus* phage M8, and one biofilm-producing strain (1/10; 10%) to *Staphylococcus* phage B4. Moreover, no plaque formation was observed in coagulase-negative *staphylococcus* (CNS) and *Escherichia coli* (*E. coli*) which indicates the specific effect of isolated phages on *S. aureus*.

### Bacteriophage latent time and burst size

**Fig. 4:**
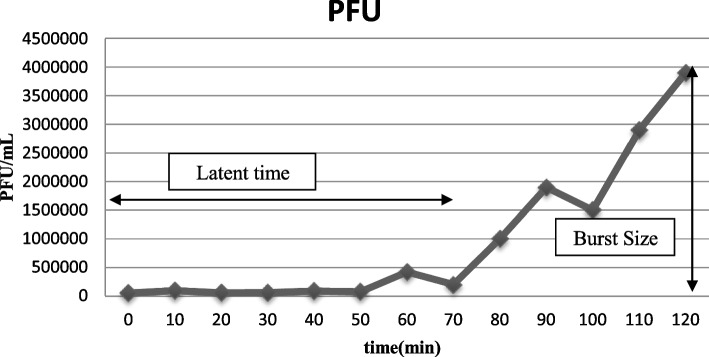
One-step growth curve of *Staphylococcus* phage M8

**Fig. 5:**
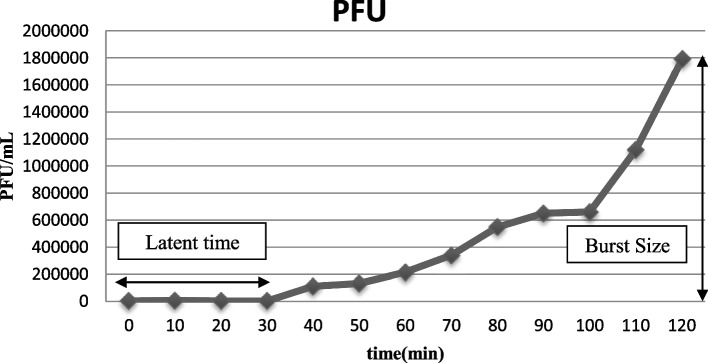
One-step growth curve of *Staphylococcus* phage B4

One-step growth experiments revealed the latent period and burst size for *Staphylococcus* phage M8 (70 min, 72 PFU/cell) and *Staphylococcus* phage B4 (30 min, 447 PFU/cell) (Figs. 4 and 5).

### Bacteriophage activity in milk

**Fig. 6:**
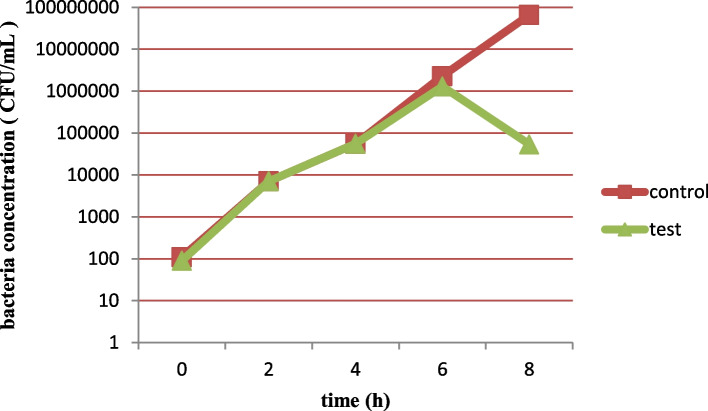
Lytic activity of *Staphylococcus* phage M8 against *S. aureus* MDR 8 at 37 °C in UHT milk

**Fig. 7:**
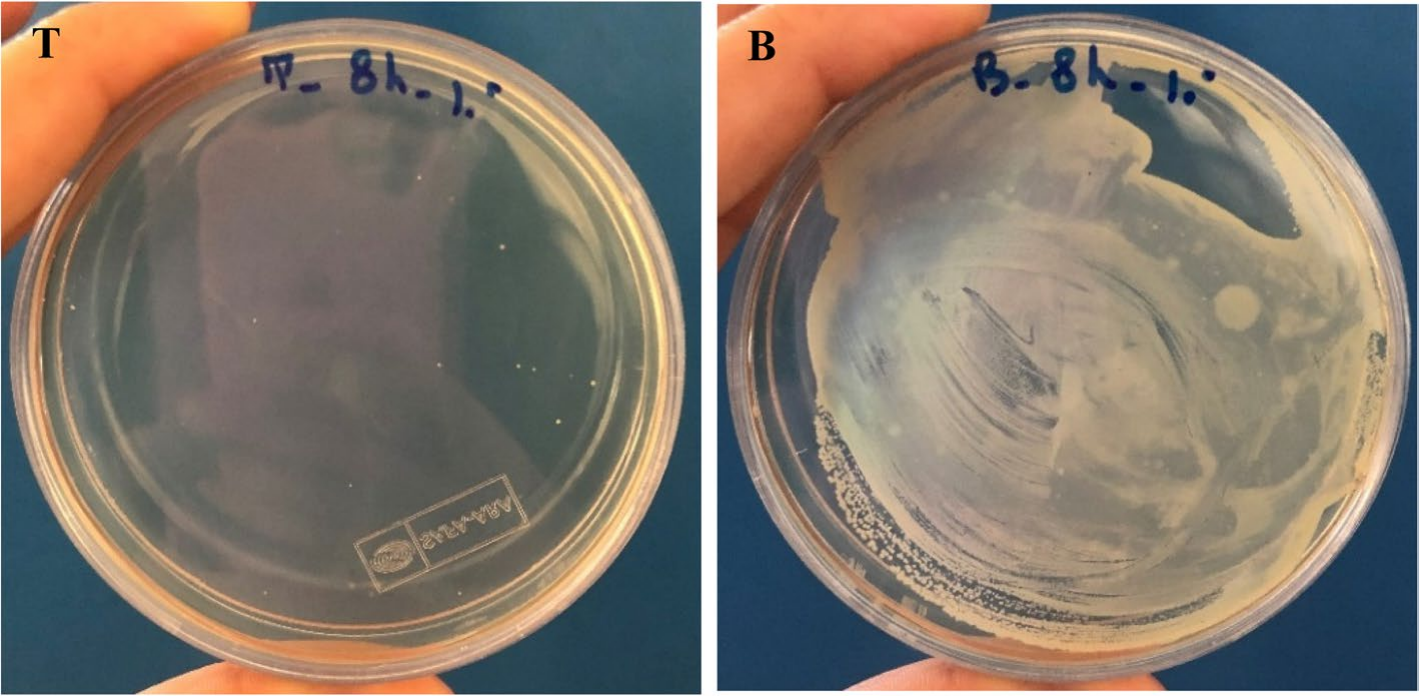
Effects of Staphylococcus phage M8 on bacterial growth in milk after 8 h of incubation. B: control (no phage); T: test (bacterial culture + *Staphylococcus* phage M8)

**Fig. 8:**
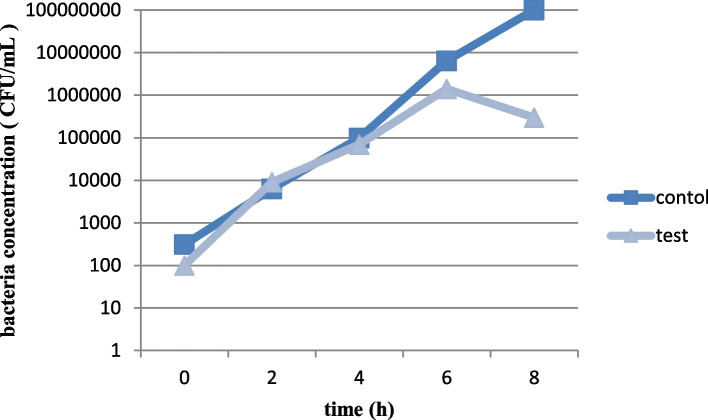
Lytic activity of *Staphylococcus* phage B4 against *S. aureus* Biofilm 4 at 37 °C in UHT milk

**Fig. 9:**
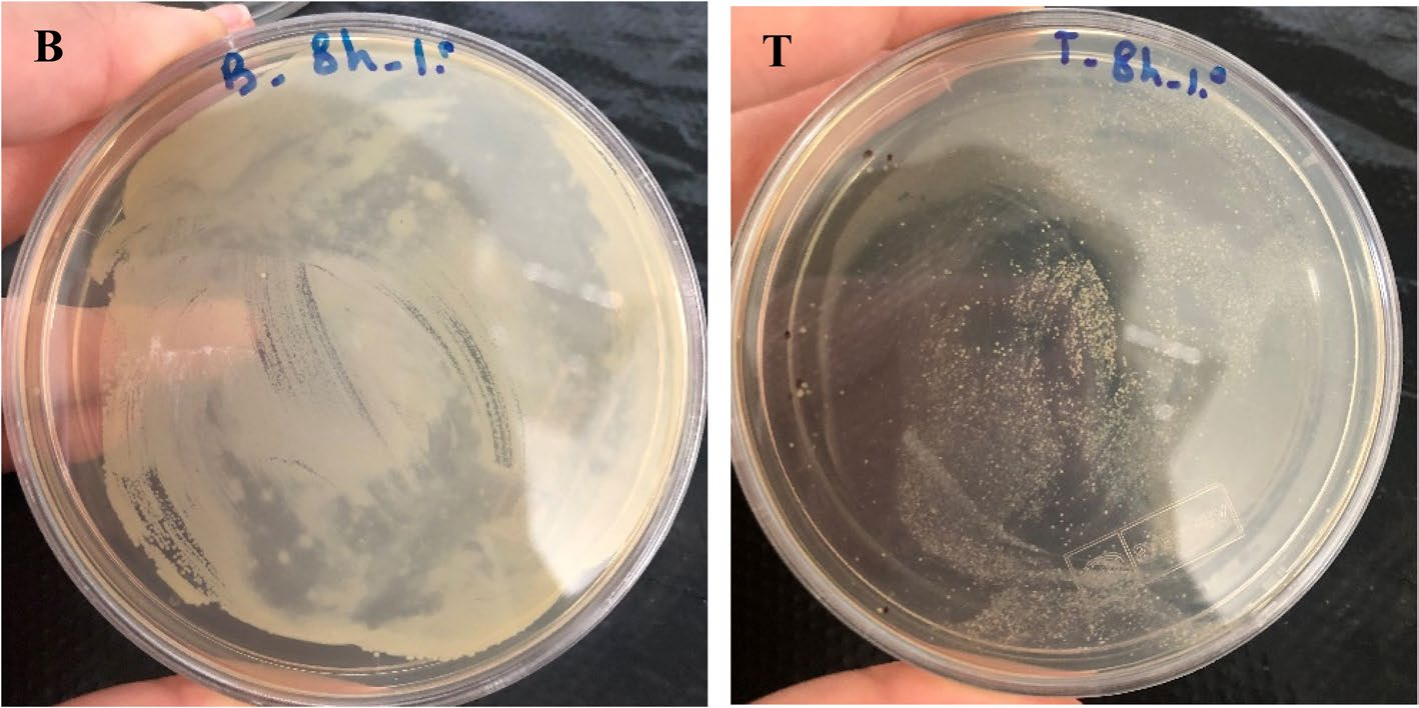
Effects of *Staphylococcus* phage B4 on bacterial growth in milk after 8 h of incubation. B: control (no phage); T: test (bacterial culture + *Staphylococcus* phage B4)

A suitable bacteriophage for therapeutic application in mastitis needs to keep its lytic activity in milk. In the present study, the activities of both bacteriophages in milk were studied which were not considerable in the first 6 h after inoculation. However, at 8 h following inoculation, bacteriophages had a clear effect on decreasing the number of bacteria in milk. Both phages were able to reduce the number of bacteria in milk at approximately 10^3^ PFU/mL (Figs. 6, 7, 8 and 9).

### Bacteriophage morphology

**Fig. 10:**
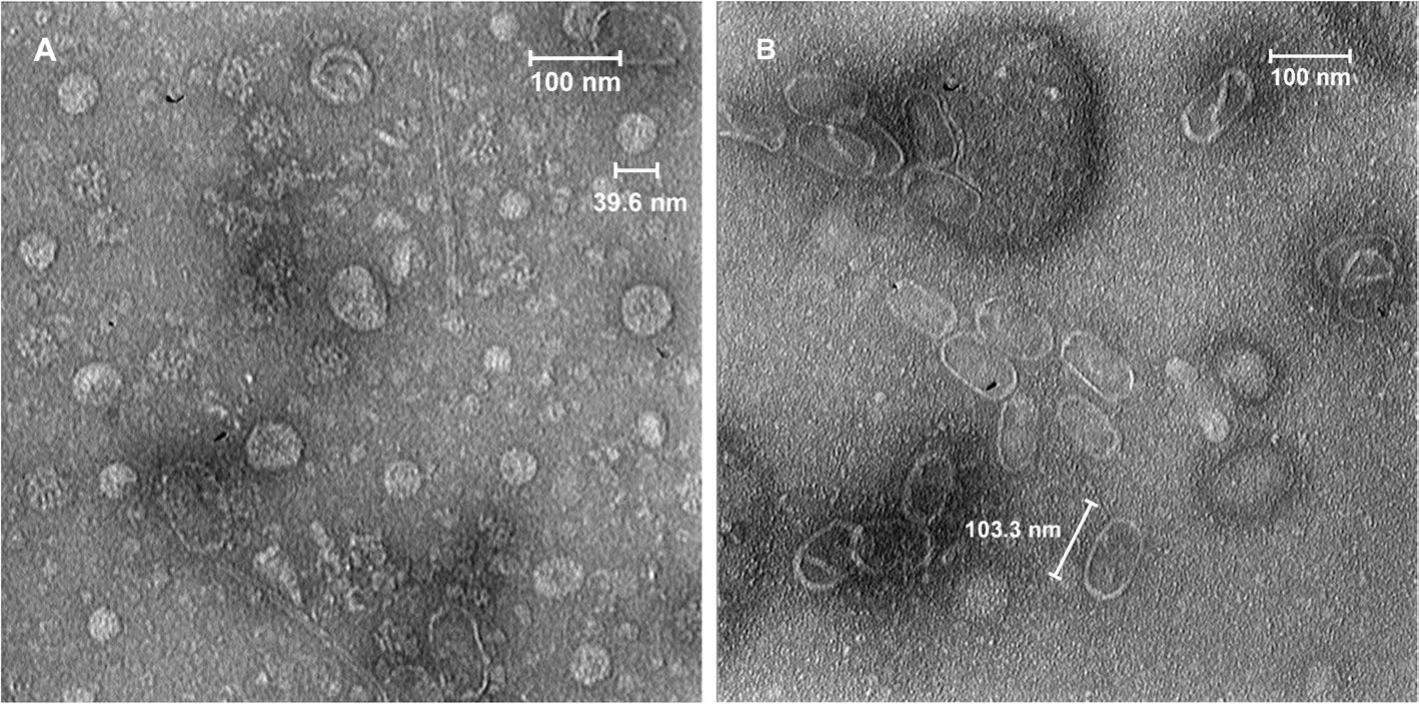
Electron microscopy image of **A**: *Staphylococcus* phage M8 and **B**: *Staphylococcus* phage B4. Phages belong to *Podoviridea* family

In the current study, both phages were classified as members of *Podoviridae* family based on their morphology determined by TEM electron microscopy (Fig. 10). Phages of this family have icosahedral capsids with short tail, or no tail at all [20]. Average size of bacteriophages M8 were 43.3 nm and average size of bacteriophages B4 were 98.1 nm.

## Discussion

*Staphylococcus aureus* is one of the most infectious pathogens causing mastitis in dairy cows. Today, the application of antibiotics in treatment of bovine mastitis caused by *S. aureus* faces serious challenges. Several pathogen-related factors are involved in the failure of specific therapeutic strategies against *S. aureus* mastitis such as long-term therapy. Rising bacterial resistance to multiple common classes of antibiotics due to mutation, resistance genes’ transfer or biofilm-formation that protect the bacterial cells against high levels of antibiotics, is one of the most concerning factors which arises a need for alternative or combinational therapeutic options [21]. Nowadays, bacteriophages are one of the most promising alternative options for affecting hard-to-treat phenotypes of *S. aureus* such as: MDR, MRSA and biofilm-producing isolates [22–24]. Most of the recent studies on bacteriophages have focused on the isolation of phages against *S. aureus* in general [18, 25, 26], and the effects of phages on MDR, MRSA and biofilm-forming strains of mastitis causing *S. aureus* have been ignored except in a few studies [13, 26, 27]. For instance, in a research conducted in 2012 by Kwiatek et al*.,* a new anti-staphylococcal phage (MSA6) was isolated from a cow with mastitis. Examination of the phage activity against 27 MRSA strains, one VRSA, 16 *S. aureus* (bovine isolated) and three *S. aureus* (human isolates) showed that phage MSA6 had a broad lytic effects on *S. aureus* strains including MRSA and mastitis causing isolates [13]. In 2013, Han et al., examined a bacterial panel consisting of 10 MRSA, 29 *S. aureus*, three *S. sciuri*, three *S. cohnii* and three *Enterococcus faecalis* against a bacteriophage SAH-1 using EOP approach. Phage SAH-1 showed a remarkable lytic activity against MRSA and other 27 *S. aureus* strains [26]. Moreover, the bacteriophage SA was introduced by Hamze et al., (2016). Examination of its lytic activity on six *S. aureus* and three MDR strains recovered from cow and buffalo showed that three *S. aureus* isolates (two MDR and one *S. aureus* strain) were susceptible to the phage SA [27]. It seems that isolation of new phages against MRSA strains is difficult [28], although in the current study, we could isolate one bacteriophage with lytic activity against MRSA strains. The efficacy of newly isolated bacteriophages (*Staphylococcus* phage M8 and *Staphylococcus* phage B4, which belong to the *Podoviridae* family) against hard-to-treat phenotypes of *S. aureus* (MDR, MRSA and biofilm-forming strains) was also investigated in the present study. According to the results, *Staphylococcus* phage M8 showed a notable lytic activity against all the tested types of *S. aureus* (MDR, MRSA and biofilm-forming strains). It seems that this bacteriophage had a potential for therapeutic applications in hard-to-treat conditions of *S. aureus* mastitis alone or in combination with other phages (phge cocktail) and antibiotics [29]. In fact, more reasearches are needed to find out these phages should exactly be combinated with and in what proportions.

Latency period and burst size are important variables when bacteriophages are considered for therapeutic purposes. Latent period and burst size for *Staphylococcus* phage M8 (70 min, 72 PFU/cell) and *Staphylococcus* phage B4 (30 min, 447 PFU/cell) were defined by one-step growth curve analysis. Han et al*.,* (2013) reported the latent period and burst size for *Staphylococcus* phage SAH-1 as 20 min and 100 PFU/cell, respectively [26]. These factors were also recorded for phages MSA6 (15 min, 23 PFU/cell) and SA (30 min, 1000 phages) [13, 27]. It is generally believed that shorter latent period along with larger burst size would be beneficial, although under some circumstances such as low density of the host bacterial cells, larger latent period would be preferred [30]. In chronic forms of *S. aureus* bovine mastitis, bacterial density is not high (usually less than 10^4^ CFU/mL) [31]. Thus, *Staphylococcus* phage M8 which has also a wider host range spectrum seems to be a potential candidate for therapeutic application. However, more comprehensive studies should be conducted to elucidate the efficacy of the isolated phages in vivo.

A suitable phage for therapeutic application in bovine mastitis needs to be active in complex media of milk as there are inhibitory components in milk which negatively affects the phage-bacteria interactions. Such inhibitory constituents which were represented by Gill et al., [32] and O’Flaherty et al*.,* [33] are high molecular weight proteins and fat globules. Phage activity in milk was evaluated by Garcia et al*.,* (2009). In the mentioned study, inhibitory activity of phages ΦA72 and ΦH5 in UHT milk on bacterial growth was examined. It was discovered that each of the phages could also inhibited *S. aureus* in milk milieu and combination of the two phages exhibited greater inhibitory effect on bacterial growth [25]. In the current study, both phages showed lytic activity by reducing the *S. aureus* population by about 3 logs in cultured milk after 8 h of incubation. However, total clearance of *S. aureus* in milk during this period was not achieved. This might be due to the low phage/cell ratio used in the experiment or because of the development of resistant strains to bacteriophages. It is widely recognized that at low cell densities, larger numbers of phages are required to ensure efficient infection of the host bacteria [25].

## Conclusion

The aim of the present study was to isolate and characterize new bacteriophages to be applicable to treatment of bovine mastitis caused by *S. aureus* that are resistant to antibiotic therapy and cannot be cured by popular prescribed classes of antibiotics. So, the target bacterial hosts of the current study were hard-to-treat phenotypes of *S. aureus* (MDR, MRSA, biofilm-producing strains) which challenge the control and therapeutic strategies of mastitis. It seems that both phages have a potential to serve as biological control agents in combination with other agents such as antibiotics. However, further studies are needed to investigate the efficacy of these phages in vivo.

## Methods

### Bacterial strains and culture conditions

In the current study, a panel of 45 bacterial isolates including 43 *S. aureus* strains (13 MRSA strains, 20 MDR strains and 10 biofilm-producing strains), one *Escherichia coli* (*E. coli*) isolate and one coagulase-negative staphylococci (CNS) isolate was chosen from bacterial collection. The non-*S. aureus* bacteria of the panel were chosen as negative controls to show the specific lytic activities of isolated phages against *S. aureus*. All the strains were obtained from bovine mastitis milk samples except MRSA strains which were isolated from burn-wound infections of human patients.

The isolates had been primarily identified by standard biochemical tests [34]. The confirmation of *S. aureus* isolates had been conducted by molecular detection of species-specific *nuc* gene [35]. In addition, molecular detection of the *mecA* gene was applied to define MRSA strains [36]. MDR strains were selected based on the definition proposed by Magiorakos et al., (2011) which introduces MDR as resistant bacteria to at least three classes of antibiotics [37]. Moreover, the ability of biofilm formation was evaluated by phenotypic method described by Peter et al., [38].

### Phage isolation and purification

For phage isolation 4 mL of *S. aureus* culture was mixed with 10 mL of fresh LB broth (2X) and 10 mL of filtered sewage sample and incubated over night at 37 °C. The mixture was then centrifuged for 20 min at 7500 × g, then the supernatant was filtered through 0.22 µm pore size filters. The lysate (supernatant) was examined for detection of the presence of lytic phages in the plaque assay by Double-layer Agar (DLA) method [39]. For this purpose, 250 microL of lysate was mixed with 150 microL of a host bacterial culture. After 10 min of incubation at 37 °C, 3 mL of LB soft agar (10 mM MgSO_4_, 0.7% (w/v) agar, at 45 °C) was added, mixed, and overlaid onto fresh LB agar plates (1.5% (w/v) agar) prepared before. After solidifying, plates were incubated overnight at 37 °C. Clear zones (plaques) over the bacterial lawn were considered as the presence of lytic phages.

For phage purification, a single plaque was picked and placed in a tube containing 1 mLof LB broth and 250 microL of host bacterial culture and incubated overnight at 37 °C. At the next day, the presence of phage was examined by DLA method. Three rounds of purification were conducted to assure about the purity of the phages. Finally, purified phages were named based on the guideline proposed by Adriaenssens and Rodney Brister (2017)[40].

### Bacteriophage titration

Ten-fold serial dilutions of bacteriophage suspensions were prepared in SM buffer (50 mM Tris–Cl, 100 mM NaCl, 8 mM MgSO_4_). Then, DLA method was applied with 100 microL of each dilution and 100 microL of host bacterial culture. Finally, phage titration (PFU/mL) was calculated by the number of plaques × 10 /dilution.

### Host range determination

Phages were screened against all the 45 bacterial strains using the efficiency of plating method (EOP) described by Wang et al*.,* [41]. EOP was calculated by dividing the titer of the phage on the tested strains by the titer of the same phage on its own isolation strain.

### One-step growth curve

One-step growth experiments were carried out with a modification of the method described by Wang et al., to determine the latent period and phage burst size [41]. Phage was added at a multiplicity of infection (MOI) of 0.01 to the cells of S. aureus and allowed to adsorb for 10 min at 37 °C. The mixture was then centrifuged at 7500 × g for 5 min. After the supernatants were removed, the pellets containing the phage-infected bacterial cells were suspended in LB Broth and incubated at 37 °C. Partial samples were obtained at 10 min intervals and the titrations from the aliquots were immediately determined using the DLA method. This assay was at least performed three times.

### Examination of bacteriophage antimicrobial activity in milk

A suitable phage for therapeutic application in mastitis needs to keep its lytic activity in complex media of milk. Therefore, the effects of isolated phages on *S. aureus* growth were evaluated in UHT milk. For this purpose, 50 microL of bacteria (10^4^ CFU/mL) was added to 5 mL of commercial UHT milk (1.5% Fat, 3% Protein) and mixed with 100 microL of bacteriophage (10^6^ PFU/mL). In parallel, a control was run containing 50 microL of bacteria (10^4^ CFU/mL) and 5 mL of UHT milk. Both of the test and control tubes were incubated at 37 °C. Then, bacterial counts were determined four times for both tubes within 8 h (with 2-h intervals) by tenfold serial dilution and double plating for each dilution [25].

### Electron microscopy

The phage containing liquid was centrifuged for 2 h at 87,000 rpm. The precipitate was then stained with 2% uranyl acetate for TEM electron microscopy (Zeiss, LEO 912 AB, Germany) at 120 kV. Moreover, the image processing program “ImageJ” was applied for measuring the phages’ structures.

## Data Availability

The datasets used or analyzed during the current study are available from the corresponding author on reasonable request.

## Abbreviations

*S. aureus*: *Staphylococcus aureus*
*E. coli*: *Escherichia coli*
CNS: Coagulase-negative staphylococci
MDR: Multidrug-resistant
MRSA: Methicillin-resistant *Staphylococcus aureus*
PFU: Plaque forming unit
CFU: Colony forming unit
SCV: Small-colony variant
EOP: Efficiency of plating
TEM: Transmission electron microscopy
DLA: Double-layer agar
MOI: Multiplicity of infection
UHT: Ultra-high-temperature
